# Identification of hot-spot regions of N-type Ca^2+^ channel receptor by homology modeling and molecular dynamics study, for structure-based blocker design

**DOI:** 10.1186/1758-2946-4-S1-P30

**Published:** 2012-05-01

**Authors:** Ashish Pandey, Satyaprakash Tripathi, C Gopi Mohan

**Affiliations:** 1 Department of Pharmacoinformatics, National Institute of Pharmaceutical Education and Research (NIPER),Sector 67, S.A.S. Nagar,Punjab -160 062, India

## 

The voltage dependent N-type Ca^2+^ channel (NCC) distributed in the nerve ending of the central and peripheral nerves. NCC is considered as potential therapeutic target for several pathological disorders like neuropathic pain and stroke disease. For understanding mechanism of action at the atomic level crystal structure provide valuable inside but lack of crystal structure of ion channel lead sequence analysis of different types of voltage dependent Ca^2+^ channel (VDCC) and we found identical/similar active site residues, which was confirmed by site-directed mutagenesis analysis of L-type Ca^2+^ channel (LCC). Based on these observations, we have developed for the first time the homology model of the closed state of NCC receptor at the ligand-sensing region by using bacterial K^+^ channel receptor. Further, molecular docking using different dihydropyridine (DHP) blockers identified NCC receptor hot spot binding residues, which is in consonance with that of the LCC. These residues are potential for further biochemical investigations. To understand binding and stability behavior of NCC with the DHP (amlodipine) in closed state, 50 nano second molecular dynamics simulation in lipid bilayer membrane environment were carried out. This analysis revealed the closed state stabilizing by binding of ligand into inner part of S6 region.

